# Medical consent; striking the right balance between shared decision-making and shared responsibility

**DOI:** 10.1093/qjmed/hcz229

**Published:** 2019-09-05

**Authors:** D S Wald, P Kelly

**Affiliations:** 1 From the Wolfson Institute of Preventive Medicine, Queen Mary University of London, London, UK; 2 Barts Heart Centre; 3 Legal Department, Barts Health NHS Trust, London, UK

It’s 8 am and the registrar has gone to consent five patients on Ward 3D, for angiograms scheduled for Catheter Lab 1 that day. The list starts at 8.30 am so the interactions are short. The doctor explains what the procedure involves, signs the consent form and asks the patient to sign and date the same form. Two of the patients speak Bengali and the form is in English. The patients have been consented.

This is a familiar, if slightly abbreviated, scenario that is not limited to cardiology. There are several learning points. First, patients are not consented; their consent is sought and they are then free to consent to the procedure. The distinction may seem semantic but the common misuse of the word consent, as a transitive verb (with a direct object), undermines its very meaning—that consent should be freely given, not done to or taken from someone.^1^

Second, consent should not, with the exception of emergency treatment, be limited to a signature on the day of a procedure. It should be a process that starts with a recommendation, is followed by information and opportunities to ask questions before consent is sought. The House of Lords judgment in Chester-v-Afshar [2004] highlights the obligation to allow patients time to reflect on information given, and more recently the High Court judgment in Thefaut-v-Johnston [2017] ruled that starting the consent process too close to surgery may invalidate consent altogether.

The General Medical Council (GMC) guidance on consent, ‘Patients and Doctors Making Decisions Together’^2^ emphasizes the need for patients to understand the benefits, risks and alternatives of a medical procedure and to encourage a dialogue that results in a shared decision between doctor and patient. The signing of a form does not demonstrate that these standards have been fulfilled. In Chatterton-v-Gerson [1981], Judge Bristow held that if a patient signs the form without understanding the procedure and risks ‘consent would have been expressed in form only, not in reality’.

## Failure to inform

In 2017, a patient in our practice developed a femoral artery complication following a coronary angiogram. He recovered but later complained he had not been informed of this risk. He spoke Bengali and whilst his wife, by his side throughout, spoke fluent English, no professional translator had been involved. An apology was made, accepted and the complaint closed. The case highlights the need to improve the process and quality of communication surrounding consent and also how exposed hospitals are to allegations of a failure to inform before consent.

**Figure 1.**
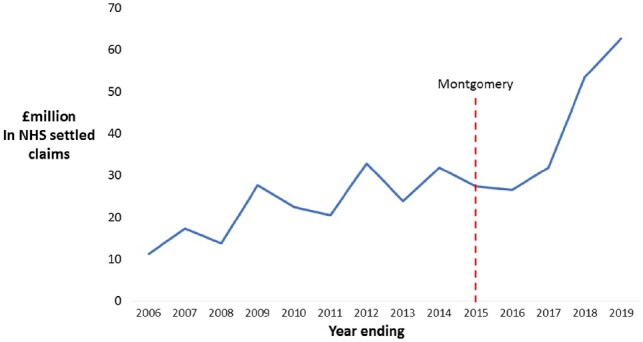
NHS payments in settled claims caused by a failure to inform before consent over time (FOI data from NHS Resolution 2019).

In the past 13 years, consent litigation costs in the NHS have increased 6-fold from about £10 million to £60 million per year (Figure 1) where a failure to inform before consent was the principal or contributory cause. The largest payments were in Orthopaedics, General Surgery and Obstetrics (about £71, £44 and £56 million, respectively). These sums reflect the tip of an iceberg, given that many complaints are not litigated and not all legal cases result in settled claims.

## Communication before consent

Written communication is often used to inform patients before consent but fewer than half read the leaflets they are given.^3^^,^^4^ A survey of 12 ophthalmology units showed that less than half the leaflets described both the benefits and risks of cataract surgery and none were judged to be understandable to an average person using a standard assessment of readability.^5^ A separate survey found that only 1 in 100 consecutive patients spent more than 5 s reading the risks of cataract surgery listed on their consent form before signing it.^6^ A fresh approach is needed.

A new digital tool, www.explainmyprocedure.com, is an online platform that hosts short animations, in different languages, describing the possible benefits, risks and alternatives of medical procedures. Patients are sent links to the animations so they can be watched at home. The animations are shown again at the pre-procedure visit, much like an airline safety video before take-off. Written consent is then recorded, about a week later, on the day of the procedure. Introduction of the animations into practice at a London centre led to a 3-fold increase in complete understanding before consent from 30 to 90%.^7^

## Montgomery standard

The Montgomery-*v*-Lanarkshire case (2015) refocused attention on informed consent. A 5-ft tall woman with diabetes, delivered her son vaginally, but complications developed due to shoulder dystocia resulting in cerebral palsy. She claimed she would have requested a caesarean section, had she known of the risks linking diabetes, large babies and small mothers. The UK Supreme Court ruled in her favour and established that in seeking consent, patients should be informed of any material risk that a reasonable person in that patient’s position might regard as significant. This may not necessarily be the same as what a body of medical practitioners think is significant, and places the test of whether reasonable care has been provided on patient opinion, rather than medical opinion (Bolam test). Doctors have expressed concern about whether this level of personalization can realistically be implemented in practice.^8^^,^^9^ Since Montgomery the number of legal pay-outs due to a failure to inform has increased sharply (Figure 1).

## Striking a balance

Neither written material nor animations will, on their own, achieve the level of personalization that the Montgomery standard sets, but such material, introduced at appropriate points in the consent process, can prompt patients to ask how a medical procedure might affect them—their particular life-styles, professions or prior medical problems. This would appropriately place some responsibility for personalizing consent on the patient. For example, a pianist may prefer to have an angiogram by the femoral artery rather than the radial artery, because of the remote chance of hand injury, even though the femoral approach is generally avoided because it increases the risk of bleeding. However, such personalization relies on patients telling doctors that they play the piano and that this is a material concern to them. The GMC emphasizes how consent should be a process of shared decision-making; it should also be one of shared responsibility.
